# A Unique Case of Melorheostosis Presenting with Two Radiologically Distinct Lesions in the Shoulder

**DOI:** 10.1155/2017/9307259

**Published:** 2017-11-06

**Authors:** Ahmed A. Elsheikh, Rohan S. Pinto, Alpesh Mistry, Simon P. Frostick

**Affiliations:** ^1^Department of Orthopaedic Surgery, Faculty of Medicine, Benha University, Benha, Egypt; ^2^North West Deanery, Manchester, UK; ^3^Department of Radiology, Royal Liverpool and Broadgreen University Hospitals, Liverpool, UK; ^4^Musculoskeletal Science Research Group, Department of Molecular and Clinical Cancer Medicine, Institute of Translational Medicine, Faculty of Health and Life Sciences, University of Liverpool, Liverpool, UK

## Abstract

Melorheostosis is a rare, nonhereditary, benign, mesenchymal condition of unknown aetiology affecting the bones and surrounding tissues. A male patient complaining of left shoulder pain, swelling, and mildly limited range of motion has an exclusive combination of the classic dripping wax lesion in the scapula and the myositis ossificans-like lesion in the deltoid muscle; this combination is the first to be reported in the shoulder. Both lesions showed typical findings of melorheostosis in radiographs, CT, MRI, and bone scan. This case has a stationary course over the follow-up period, and no specific treatment is needed in due course.

## 1. Introduction

Melorheostosis is a rare, nonhereditary, benign, mesenchymal condition of unknown aetiology affecting the bones and surrounding tissues [1]. The incidence is not truly known but has been estimated to be 0.9 per 1,000,000 [2]. The etymology of melorheostosis derives from the Greek terminology—*melos* [limb], *rhein* [flow], and *osteos* [bone] [3]. It is characterized by cortical bone thickening resulting in irregular hyperostosis that appears to flow down the length of the bone [4]. Radiologically, the appearances are often compared with dripping candle wax, and for this reason, the condition is sometimes referred to as candle disease of the bone [5]. We present a unique case of melorheostosis presenting with two radiologically distinct lesions in the shoulder joint.

## 2. Case Report

A 51-year-old male presented to our clinic with a painful swelling in his left shoulder. The pain started gradually ten months before presentation and was progressive in nature. There was no history of trauma. The swelling had been gradually increasing in size over a period of three weeks after which growth became static. The pain was provoked by activities requiring shoulder elevation and abduction. Shoulder rotation did not provoke pain. The patient has no clinically relevant past medical or family history.

On examination, a swelling was identified in the antero-superior aspect of the left shoulder with redness of the skin overlying it. The swelling was firm, localised, and mildly tender. The patient demonstrated almost full range of motion with mild pain in abduction and internal rotation (impingement and the Hawkins test were positive) with negative tests for biceps tendonitis, rotator cuff tear, AC joint arthritis, and instability.

**Figure 1:**
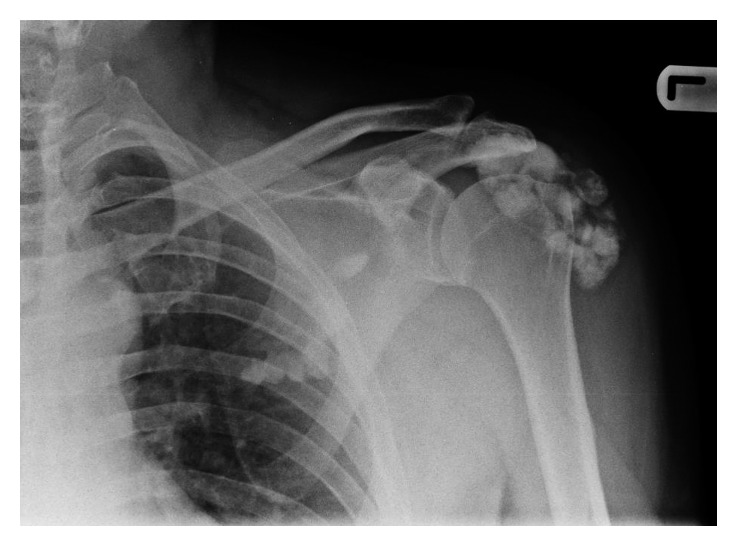
Anteroposterior radiographs of the left shoulder showing calcification over the greater tuberosity and subacromial space as well as classic hyperostotic lesions in the scapular blade.

**Figure 2:**
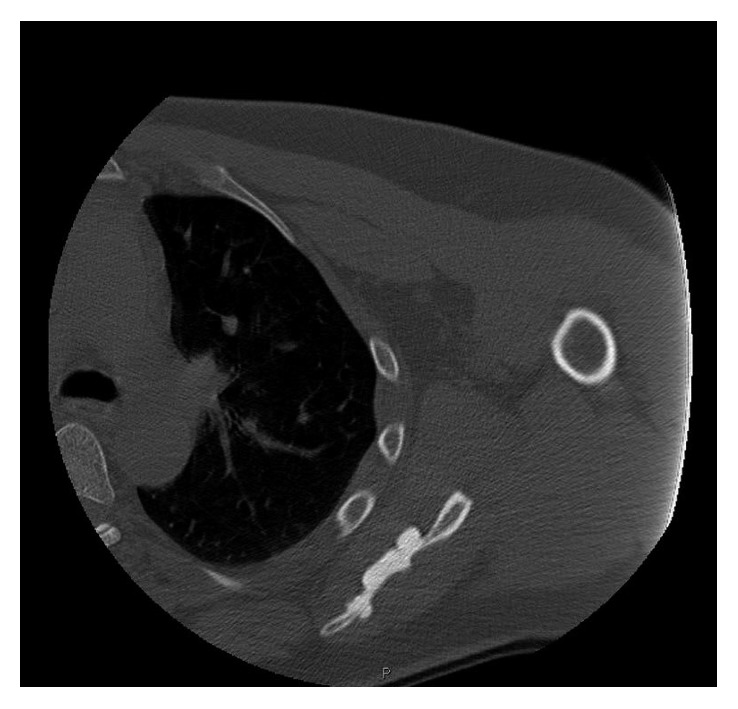
Computed tomography scan of the left shoulder. Axial cut showing hyperostotic masses dribbling from the scapular blade.

**Figure 3:**
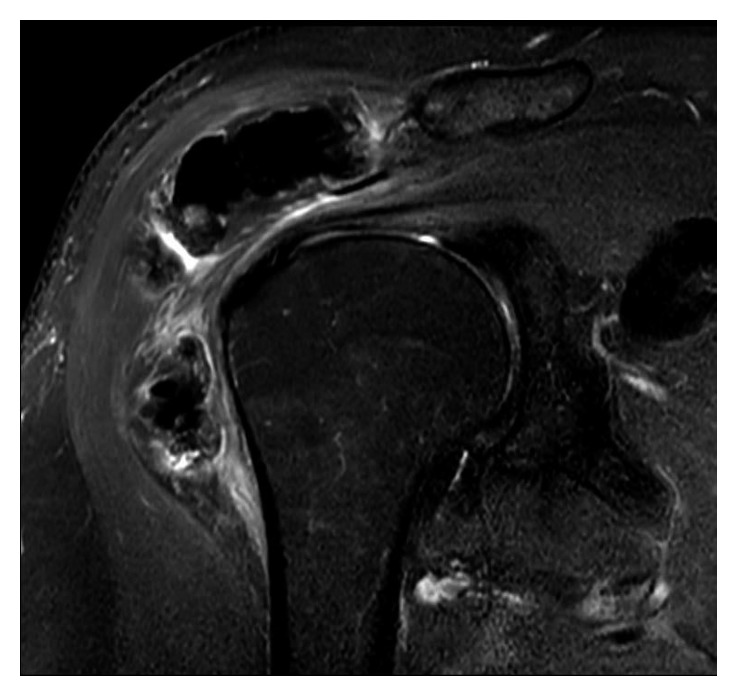
Magnetic resonance imaging of the left shoulder. Coronal T2 fat suppression image showing low-signal intensity calcific foci within and beneath the deltoid muscle with no intra-articular involvement.

Radiographs demonstrated calcification around the greater tuberosity and subacromial space as well as hyperostotic lesions in the scapula blade (Figure 1). A CT scan revealed extensive yet well-defined ossification within the proximal deltoid muscle and hyperostotic masses dribbling from the scapular blade (Figure 2). An MRI scan showed a large volume of low-signal intensity calcific foci within and beneath the deltoid muscle with no intra-articular involvement or extension to the proximal humerus itself. There were also multiple areas of cortical thickening of the scapula (Figure 3). Otherwise, all structures are normal. Tc-99m MDP bone scan revealed increased uptake in the lesions in the left shoulder, indicating high-grade osteoblastic activity. No other site of abnormal tracer activity was identified. These findings are consistent with isolated melorheostosis localised to the shoulder region.

## 3. Discussion

Melorheostosis was described for the first time in 1922 by Leri and Joanny [6]. It is characterized by hyperostotic linear bone densities and soft tissue contractures and ossification [7]. So far, 313 and 223 cases have been reported in the international and Chinese literature, respectively [5]. The reported age range of presentation for melorheostosis is between 1 and 63 years of age [8], which is consistent with our patient's age. Our patient presented with a monomelic distribution in his left shoulder. There are conflicting reports about the prevalence of monomelic versus polyostotic distributions of melorheostosis [5, 8]. However, it is clear that lower limb involvement is more common than upper limb, rib, and spinal involvement [5, 8] and that the hands [7] are more frequently affected than the shoulder in upper limb cases [3, 5, 8–13].

Several theories to explain the pathogenesis of melorheostosis have been proposed. In 1979, Murray and McCredie [14] suggested that an early embryonic abnormality of a spinal sensory nerve affecting a single sclerotome resulted in bony overgrowth. This theory is consistent with our patient who has a left scapular blade lesion confined to the C6 sclerotome as well as with many other cases reported in the literature. It has been suggested that the skin and soft tissue involvement seen in many cases may result from trauma to the corresponding dermatome or myotome [14]. In 1995, Fyns hypothesised that mosaicism was responsible for the development of melorheostosis involving a postzygotic mutation of the mesenchyme resulting in concomitant bony, cutaneous, vascular, and soft tissue involvement. Surprisingly, the soft tissue ossification with the redness in the skin and the bony lesion of the scapula in the shoulder of the presented case can be easily explained by this theory [15].

Some studies have attempted to investigate the genetic origin and inheritance pattern of melorheostosis [16]. The *LEMD3* gene which encodes for the inner nuclear membrane protein is responsible for controlling bone growth, and mutations in this gene can lead to a proliferation of hyperostotic lesions. Germline *LEMD3* mutations were found in patients who had melorheostosis associated with Buschke-Ollendroff syndrome (BOS) or Osteopoikilosis [17, 18]. However, *LEMD3* mutations have never been identified from lesional tissue from isolated cases of melorheostosis [18].

Our patient presented with shoulder pain, which is the most common presenting complaint documented in the literature [5, 8]. However, many patients are diagnosed based on incidental radiological findings [1, 13]. Other reported signs and symptoms include stiffness and reduced range of movement of the affected joint [11, 12, 19], soft tissue contractures or masses [3, 10, 20], various skin manifestations [3], and bone shortening and deformity [7–9, 12, 21]. Carpal tunnel syndrome is a rare presentation of melorheostosis [22].

The diagnosis of melorheostosis is usually established through radiological findings: X-rays, computed tomography, magnetic resonance imaging, and bone scans; specific features have been described in every modality; and these findings gave a solid foundation for diagnosis in many studies [1, 8, 11, 13, 16, 17, 19, 21–25]. A biopsy was carried out in cases of sinister or suspicious lesions and in many incidences as part of the surgical intervention [5, 7–10, 12, 18, 20, 26, 27], but this was not mandatory for every case.

Melorheostosis in adults has four distinct radiological appearances in the X-ray: the classic dropping wax appearance, osteoma-like lesion, myositis ossificans- (MO-) like lesion, osteopathia striata-like lesion, and mixed picture [3]. Children with melorheostosis have different radiological pictures [8]. To our knowledge, our case has a unique combination of a classic lesion in the scapular blade and a myositis ossificans-like lesion in the deltoid muscle. The typical hyperostotic lesion is present in most cases involving the shoulder [1, 9–13]. However, no published cases are reported on the combination seen in our patient [5, 8]. Differentiating between MO and MO-like melorheostosis lesions is essential. Trauma usually precedes MO, and nodular calcification is seen in radiological studies for melorheostosis rather than the original lamellar pattern [3, 16]. Furthermore, a bone scan of a patient with melorheostosis will demonstrate a significantly higher tracer uptake than that would be seen in a patient with MO [28].

Computed tomography [CT] and magnetic resonance imaging [MRI] scans have a supporting role in diagnosing melorheostosis, CT commonly shows high attenuation cortical thickening occluding the medulla, and clear linear demarcation is seen between the lesion and the healthy bone. Soft tissue lesions are easily identified on CT. A degree of mineralisation is seen, and often the soft tissue lesions are not in continuity with the bone [10, 16]. The CT scan of our patient demonstrated this classic presentation.

MRI of bone lesions in melorheostosis shows low signal intensity on all pulse sequences encroaching on the medullary canal which is typical for the scapular lesion of the case presented. Soft tissue lesions produce heterogeneous MRI patterns according to the degree of mineralisation. Low signal intensity is detected in mineralised lesions as is shown in our case. Intermediate to high signal is predominantly in the nonmineralised lesions [10, 16, 20].

Since 1976, Tc-99m bone scintigraphy has been developed as a tool to confirm the diagnosis of melorheostosis and unveil other silent lesions [29, 30]. Melorheostosis causes increased tracer uptake, which bridges over the joints due to hypervascularity, which has been confirmed by angiographic studies [16, 28]. The reduced tracer uptake observed in patients treated with bisphosphonates supports the theory that increased osteoblastic activity and turnover are key processes occurring in melorheostosis [9, 23].

In this case, based on our findings, we concluded that this is a case of isolated melorheostosis. The common differential diagnosis of melorheostosis includes myositis ossificans, synovial osteochondromatosis, osteoma, parosteal osteosarcoma, focal scleroderma, and Caffey disease. Combined clinical examination and full radiological workup can accurately differentiate diagnoses. Osteoma presents with a smooth outline, focal, single vertebral involvement in the spine. Caffey disease affects infants with lamellated periosteal reaction, transient and less dense. Classic cauliflower-like ossified mass characterises parosteal osteosarcoma, a lucent line between lesion and cortex, increased uptake on bone scan. Osteopathia striata may be mistaken for melorheostosis, but striations in melorheostosis are much larger, broader, and unilateral, unlike the genuine osteopathia striata. Osteopoikilosis has numerous round to ovoid white densities of similar size spread throughout all bones; approximately 10% of osteopoikilosis is accompanied by skin elastic or collagen nevi, named as BOS. Tumor cacinosis is usually associated with a systemic disorder of calcium metabolism or renal dialysis, presents bilaterally and causes erosion of cortex, amorphous and cystic multilobulated calcification in periarticular distribution. Calcium pyrophosphate dihydrate deposition (CPPD) disease affects the elderly; with a characteristic affinity to ligamentum flavum and synovial joints of the spine is in ligamentum flavum and synovial joints [7, 19].

There is no standard treatment for melorheostosis, and management plans must be made on an individual patient basis [8]. The aims of treatment are pain relief and maintaining function. There are a few reports describing successful analgesia with the use of bisphosphonates [9, 23]. Surgical procedures may be offered to patients experiencing mechanical symptoms such as nerve compression, contractures, impingement, and deformity [7, 12, 21, 22, 27].

We have followed our patient over six months, and radiologically, the findings are stationary. Clinically, pain has settled down, and he has nearly no mechanical symptoms, thus no rationale for any intervention in the meantime.

## 4. Conclusion

Our patient has a unique presentation of melorheostosis presenting with two distinct coinciding lesions in the shoulder joint. Our case supports existing theories regarding the aetiology of the disease and contributes to the literature on the spectrum of possible presentations of melorheostosis.
